# Regenerative and Protective Actions of the GHK-Cu Peptide in the Light of the New Gene Data

**DOI:** 10.3390/ijms19071987

**Published:** 2018-07-07

**Authors:** Loren Pickart, Anna Margolina

**Affiliations:** R&D Skin Biology; 4122 Factoria Boulevard SE, Suite Number 200, Bellevue, WA 98006, USA; drlorenpickart@gmail.com

**Keywords:** GHK, GHK-Cu, gene profiling, wound healing, COPD, skin regeneration, anti-oxidant, fibrinogen

## Abstract

The human peptide GHK (glycyl-l-histidyl-l-lysine) has multiple biological actions, all of which, according to our current knowledge, appear to be health positive. It stimulates blood vessel and nerve outgrowth, increases collagen, elastin, and glycosaminoglycan synthesis, as well as supports the function of dermal fibroblasts. GHK’s ability to improve tissue repair has been demonstrated for skin, lung connective tissue, boney tissue, liver, and stomach lining. GHK has also been found to possess powerful cell protective actions, such as multiple anti-cancer activities and anti-inflammatory actions, lung protection and restoration of chronic obstructive pulmonary disease (COPD) fibroblasts, suppression of molecules thought to accelerate the diseases of aging such as NFκB, anti-anxiety, anti-pain and anti-aggression activities, DNA repair, and activation of cell cleansing via the proteasome system. Recent genetic data may explain such diverse protective and healing actions of one molecule, revealing multiple biochemical pathways regulated by GHK.

## 1. Introduction

The human copper-binding peptide GHK-Cu (glycyl-l-histidyl-l-lysine) is a small, naturally occurring tri-peptide present in human plasma that also can be released from tissues in case of an injury. Since its discovery in 1973, GHK-Cu established itself as a powerful protective and regenerative ingredient, which is currently widely used in skin and hair products [1].

Up-to-date, it is established that GHK-Cu is able to:

- Tighten loose skin and reverse thinning of aged skin
- Repair protective skin barrier proteins
- Improve skin firmness, elasticity, and clarity
- Reduce fine lines, depth of wrinkles, and improve structure of aged skin
- Smooth rough skin
- Reduce photodamage, mottled hyperpigmentation, skin spots and lesions
- Improve overall skin appearance
- Stimulate wound healing
- Protect skin cells from UV radiation
- Reduce inflammation and free radical damage
- Increase hair growth and thickness, enlarge hair follicle size

Most authors would attribute effects of GHK to its ability to bind copper(II) ions. It was proposed that because of the GHK’s small size and its ability to bind copper, it can play a crucial part in copper metabolism [2]. However, since 2010, a new mechanism has started to emerge. The Broad Institute of MIT and Harvard (Cambridge, MA, USA) has created the Connectivity Map—a publicly available library of transcriptional responses to known perturbagens, substances that modulate gene expression [3]. This tool allowed researchers to investigate genome-wide effects of GHK and establish that GHK-Cu is able to up- and down-regulate a significant number of human genes. Today, it has become possible to connect biological effects of GHK-Cu and its effects on gene expression, to develop a more comprehensive view on GHK’s mechanism of action [4].

The present paper reviews protective and regenerative actions of the GHK-Cu peptide in human skin, as well as new gene data, revealing possible mechanisms behind these actions.

## 2. GHK and Gene Expression

The number of human genes stimulated or suppressed by GHK with a change greater than or equal to 50% is 31.2%. GHK increases gene expression in 59% of the genes, while suppressing it in 41%. For our studies, we used the gene expression results from 50% . This gave the best correlation with our biological data. Table 1 presents an estimate of the number of genes affected by GHK at various cutoff points.

### 2.1. GHK Improves Skin Regeneration

Skin’s ability to withstand damage and repair itself is highest in children and young individuals because of well-functioning repair and protective mechanisms. However, with age, skin’s ability to repair damage declines. GHK content is highest in the plasma of young, healthy individuals. At age 20, the plasma level of GHK is about 200 ng/mL (10^−7^ M), and by the age of 60, it declines to 80 ng/mL. In the experiment that led to discovery of GHK, plasma from young individuals added to liver tissue obtained from older individuals, caused old liver tissue to produce proteins more characteristic of younger individuals [6].

In the 1980s, Maquart et al. proposed that GHK may be an early signal for skin repair. The GHK amino acid sequence is present in the alpha 2(I) chain of type I collagen, and when damage activates proteolytic enzymes, GHK is released into the site of an injury [7]. A number of experiments established that GHK stimulates synthesis of collagen, selected glycosaminoglycans and small proteoglycan decorin [8,9]. It also modulates activity of key metalloproteinases, which are enzymes that facilitate breakdown of proteins of extracellular matrix, as well as activity of anti-proteases. This suggests a general regulatory effect on protein breakdown in skin, helping to prevent both buildup of damaged proteins and excessive proteolysis [10,11]. Since excessive breakdown of the dermal matrix as well as inadequate removal of damaged proteins can negatively affect skin’s health and appearance, GHK’s ability to regulate both metalloproteinases and their inhibitors can support skin regeneration and improve its appearance.

GHK also demonstrated beneficial effects on skin fibroblasts, which are considered key cells in the skin regeneration process. Fibroblasts not only synthesize structural elements of the dermal matrix but also produce a wide range of growth factors essential for skin repair. GHK, in combination with LED irradiation (light emitting diode irradiation, 625–635 nm), compared with the LED irradiation alone increased: cell viability 12.5-fold, production of the basic fibroblast growth factor (bFGF), 230%, and collagen synthesis, 70% [12].

GHK-Cu has been found to stimulate epidermal basal cells, markedly increasing integrins and p63 expression. The cells’ shape became more cuboidal, which indicates an increase in their stemness [13].

### 2.2. Cosmetic Use of GHK-Cu

A number of clinical studies confirmed GHK-Cu’s ability to improve appearance of aging skin. A facial cream containing GHK-Cu applied for 12 weeks to the facial skin of 71 women with mild to advanced signs of photoaging increased skin density and thickness, reduced laxity, improved clarity, reduced fine lines and the depth of wrinkles [14].

A GHK-Cu eye cream applied for 12 weeks to around-the-eye area of 41 women with mild to advanced photodamage performed better than placebo and vitamin K cream. It reduced lines and wrinkles, improved overall appearance, and increased skin density and thickness [15].

GHK-Cu applied to thigh skin for 12 weeks improved collagen production in 70% of the women treated, in contrast to 50% treated with the vitamin C cream, and 40% treated with retinoic acid [16]. In addition to improving skin laxity, clarity, firmness and appearance, reducing fine lines, coarse wrinkles and mottled pigmentation, and increasing skin density and thickness, GHK-Cu cream applied twice daily for 12 weeks also strongly stimulated dermal keratinocyte proliferation [17].

With their pilot study for topical application of copper tripeptide complexes in aged skin, Krüger et al. confirmed an increase in skin thickness in the range of the epidermis and dermis, improved skin hydration, a significant smoothing of the skin by stimulating collagen synthesis, increased skin elasticity, a significant improvement in skin contrast and an increased production of collagen I [18,19].

GHK-Cu at 0.01, 1 and 100 nM incubated with human adult dermal fibroblasts increased production of elastin and collagen. GHK also increased gene expression of MMP1 and MMP2 at the 0.01 nM. All concentrations increased TIMP1. The effects of GHK-Cu were also investigated in a randomised, double–blind clinical trial. Female volunteers applied GHK-Cu, encapsulated in nano-lipid carrier twice a day in the course of 8 weeks using either carrier alone or the commercially available peptide Matrixyl^®^ 3000 as controls. Compared to Matrixyl^®^ 3000, GHK-Cu produced a 31.6% reduction of wrinkle volume. Compared to control serum, GHK-Cu reduced wrinkle volume 55.8% and wrinkle depth 32.8% [20].

### 2.3. Animal Studies Confirm Wound Healing Activity of GHK

Multiple animal studies have established the wound healing activity of GHK. It appears that GHK stimulates wound healing through a variety of mechanisms. In rabbit experimental wounds, GHK alone or in combination with high dose helium–neon laser improved wound contraction and formation of granular tissue, as well as increasing activity of antioxidant enzymes and stimulating blood vessel growth [21,22]. Collagen dressing with incorporated GHK (PIC-Peptide Incorporated Collagen) accelerated healing of wounds in healthy and diabetic rats. The treated group displayed higher glutathione (GSH) and ascorbic acid levels, better epithelialization, as well as increased synthesis of collagen and activation of fibroblasts and mast cells in wounds. In healthy rats, treatment of wounds with PIC increased collagen 9-fold [23,24]. GHK-Cu improved healing of ischemic open wounds in rats. Wounds displayed faster healing, decreased concentration of metalloproteinases 2 and 9 as well as of TNF-β (a major inflammatory cytokine) compared with vehicle alone or with untreated wounds [25].

One problem with GHK-Cu is that it is very sensitive to breakdown by carboxypeptidase enzymes. Wounds such as diabetic skin ulcers or bedsores usually develop a “wound serum”, thought to be generated by airborne bacteria settling on the wound. The “serum” rapidly breaks down GHK and probably other growth factors such as TGF (Transforming Growth Factor) and PDGF (Platelet Derived Growth Factor).

### 2.4. Stimulation of Blood Vessel and Nerve Growth

Nerve and blood vessel growth is an important factor in skin healing and regeneration.

Sage et al. observed that GHK and related peptides are produced in the course of protein breakdown after an injury from a SPARC protein. SPARC (Secreted Protein Acidic and Rich in Cysteine) is a glycoprotein, mostly expressed in embryonic tissues and in tissues undergoing repair and remodeling. At initial stages of tissue repair, GHK and other peptides containing the GHK sequence (such as KGHK), which are released from SPARC in the course of proteolysis, stimulate new vessels growth. Later in the healing process, GHK and GHK-related peptides inhibit blood vessel growth [26].

#### Promotion of Nerve Outgrowth

When skin healing is inadequate, the healed area is often devoid of sensory abilities. In cell cultures, both Monique Sensenbrenner’s lab (France) and Gertrude Lindler’s lab (Germany) found that GHK stimulates nerve outgrowth, an essential attribute of skin repair. GHK helps restore skin’s innervation through increased production of neurotrophic factors [27,28].

Ahmed and colleagues at the Neurochemistry Lab in Chennai, India wrote that when severed nerves within a rat are placed in a collagen tube impregnated with GHK, there is an increased nerve outgrowth. GHK-Cu increased production of nerve growth factor and the neurotrophins NT-3 and NT-4, sped up the regeneration of nerve fibers from nerve stubs placed in a collagen tube, and increased axon count and proliferation of Schwann cells compared to the control group [29].

When we searched for GHK’s gene activation effects on the Gene Ontology for neurons, we came up with 408 genes up and 230 genes down. So GHK has a significant effect on neurons, but we don’t know exactly what this means. With time, we will be able to analyze the huge amount of data. Table 2 presents the top 10 genes upregulated by GHK and the top 10 downregulated [30].

### 2.5. Anti-Oxidant and Anti-Inflammatory Actions

As animal experiments show, treatment of wounds with GHK leads to elevated levels of antioxidant enzymes. GHK also possesses strong antioxidant and anti-inflammatory actions. GHK inactivated damaging free radical by-products of lipid peroxidation, such as 4-hydroxynoneal, acrolein, malondialdehyde, and glyoxal, protecting cultured skin keratinocytes from ultraviolet (UV)-radiation [31]. GHK was shown to completely block Cu(2+)-dependent oxidation of low density lipoproteins (LDL). Another well-known anti-oxidant, which is also widely used in skin care, superoxide dismutase (SOD1), gave only 20% protection [32]. GHK also prevents damaging effects of lipid peroxidation, by binding its by-products such as acrolein and 4-hydroxynonenal [33,34].

GHK:Cu(2+) reduced iron release from ferritin by 87%. Ferritin in blood plasma can store up to 4500 atoms of iron per protein molecule, which is a well-known catalyst of lipid peroxidation—a chain reaction, which produces a slew of free radicals, leading to DNA, protein and cell membrane damage. Disturbances in iron metabolism contribute to many pathological conditions, including brain damage and neuron death under various neurological conditions. When iron is released from ferritin, it can form an Fe(2+)/Fe(3+) complex and start the chain reaction of lipid oxidation [35].

### 2.6. Lung COPD and Acute Lung Injury

Successful tissue regeneration requires collaboration of multiple cells, which is orchestrated by various cytokines and growth factors. This means that in order for regeneration to be successful, these molecules have to be produced and released in the right amount and in the right place. This is important because no signal molecule works on its own, but instead engages in a crosstalk, which leads to activation of certain cellular pathways. Among pathways involved in skin regeneration are cellular pathways regulated by TGF-β and integrins [36].

GHK appears to support remodeling and restructuring of connective tissue, modulating expression of numerous genes, including up-regulation of genes of the TGF-β pathway. This is evident from a study which demonstrated GHK’s ability to reverse expression of key genes, included in a gene signature of COPD—Chronic Obstructive Pulmonary Disease. The expression of 127 genes was altered in COPD patients. More severe emphysema symptoms were correlated with the degree of change in gene expression. Genes whose expression was associated with inflammation were upregulated, and genes involved in tissue remodeling and repair were downregulated significantly. Using the Connectivity Map, a software gene profiling tool developed by the Broad Institute, the researchers sorted through gene expression profiles of biological molecules and came up with GHK as a compound which could reverse changes in gene expression associated with emphysematous destruction, such as decreased activity of genes involved in the TGF-β pathway. GHK was able to change the gene expression pattern to its opposite—activation of the TGF-β pathway.

GHK was then tested in vitro to confirm its positive effects on connective tissue. Lung fibroblasts from COPD patients, which had impaired ability to contract and restructure collagen, were treated with GHK or TGF-β. Both molecules restored function of fibroblasts. They also had an elevated expression of integrin beta 1 [37,38].

The use of GHK-Cu in mice protected their lung tissue from induced acute lung injury (ALI) and suppressed the infiltration of inflammatory cells into the lung. The GHK-Cu also increased superoxide dismutase (SOD) activity while decreasing TNF-1 and IL-6 production through the blocking activation of NFκB’s p65 and p38 MAPK (mitogen activated protein kinase). Mitogen activated protein kinases are kinase enzymes that play crucial part on cellular signaling. P38 MAPK pathways enable cells to respond to a wide range of external stressors, and affect skin differentiation, apoptosis, mobility and gene expression. NFκB p65 activation has been found to be correlated with many diseases of aging and cancer development [39].

### 2.7. Blocking of Cortisone Effects

Topical steroids, such as cortisone, are often prescribed for treatment of inflammatory cutaneous disorders, can inhibit wound repair, as well as produce skin thinning and other defects [40].

GHK-Cu, administered systemically to mice, rats, and pigs, has protective effects on cortisone—induced inhibition of wound healing [41].

### 2.8. Suppression of Fibrinogen

GHK was isolated as a plasma molecule that suppressed fibrinogen. Fibrinogen is best known for its ability to form blood clots. However, it also heavily influences the flow of blood through the microcirculation, where blood acts as a thixotropic fluid, somewhat like toothpaste, flowing under increased pulses of blood pressure, then reverting to a semi-solid gel. Elevated fibrinogen greatly increases the blood viscosity in the microcirculation by increasing red blood cell "stacking" or rouleaux formation. Studies in Germany and Scotland have found that fibrinogen levels are the top risk factor for cardio vascular diseases (CVD). The Prospective Cardiovascular Münster (PROCAM) study followed 5389 men for 10 years. It found that the incidence of coronary events in the top third of the plasma fibrinogen levels was 2.4-fold higher than in the bottom third. Individuals in the top third of levels of low-density lipoprotein (LDL) cholesterol who also had high plasma fibrinogen concentrations had a 6.1-fold increase in coronary risk. Unexpectedly, individuals with low plasma fibrinogen had a low incidence of coronary events even when serum LDL cholesterol was high [42].

The Scottish Heart Health Study followed 10,359 men and women for 2 years, and fibrinogen was the single most powerful risk factor for CVD risk or death and more predictive than lipoprotein cholesterol. The increase in (relative risk) between the highest and lowest fibrinogen levels was 301% for men and 342% for women (CVD death) and 259% for men and 220% for women (death from any cause) [43].

As mentioned above (Park et al.), GHK suppresses the production of Interleukin-6, a main positive regulator of fibrinogen production, both in cell cultures and in mice. As our gene profiling data indicate, GHK downregulates (−475%) the gene for the beta chain of fibrinogen. Since equal amounts of all three polypeptide chains are needed to produce fibrinogen, when synthesis of one of the chain of fibrinogen is suppressed, it will have a general inhibitory effect on fibrinogen synthesis [44].

### 2.9. Skin Remodeling and Anti-Cancer Actions

A major concern for any substance, which activates cell growth and tissue remodeling—is whether it can also trigger cancer. Therefore, it is very important to notice that GHK, which repairs skin, also possesses potent anti-cancer properties. 

In 2010, Hong et al. used Broad Institute’s Connectivity Map to find molecules that could inhibit metastatic colon cancer. The Connectivity Map contains expression profiles that were evaluated in five cancer cell lines, in response to 1,309 bioactive small molecules. A search through the database produced only two substances that were able to down-regulate expression of “metastatic” genes— two skin remodeling substances, GHK and the plant alkaloid, securinine. GHK produced the result at a low non-toxic 1 micromolar concentration and securinine at 18 micromolar. GHK suppresses RNA production in 70% of 54 human genes overexpressed in cancer patients, including “node molecules” YWHAB, MAP3K5, LMNA, APP, GNAQ, F3, NFATC2, and TGM2. These molecules play key roles in regulation of important molecular pathways [45]. This shows that GHK is involved in gene regulation of various biochemical pathways, and it seems to be resetting the gene activity back to health, which leads to the improvement of tissue repair [46].

UV-radiation and other damaging factors can damage skin cells’ DNA, which can potentially lead to skin cancer. One of the main protective mechanisms is apoptosis or programmed cell death. Normal healthy skin cells have checkpoint systems to self-destruct if they are synthesizing DNA incorrectly. When apoptosis is inhibited, skin cancer risk greatly increases. Also, apoptosis is the mechanism through which many anti-cancer treatments, including melanoma treatments, work. Some common cosmetic ingredients, such as elderberry extract, can enhance effectivity of cancer treatment by enhancing apoptosis in malignant cells [47].

Matalka et al. demonstrated that GHK, at 1 to 10 nM, inhibited the growth of human SH-SY5Y neuroblastoma cells and human U937 histiocytic lymphoma cells. It also re-activated the apoptosis system, as measured by the caspases 3 and 7. In contrast, GHK stimulated the growth of healthy human NIH-3T3 fibroblasts [48].

In 1983, using a method developed by Linus Pauling’s group [5], we tested the mixture of GHK-copper 2+ plus ascorbic acid (vitamin C) on the growth of sarcoma-180 in mice. This gave a very strong suppression of the cancer. These results remained unpublished until 2014, when we could supplement these findings with gene data. We used the Broad Institute Connectivity Map to investigate the effect of GHK on genes relevant to cancer growth. GHK upregulated the expression of 10 caspase and caspase-associated genes. It also affected 84 genes associated with DNA repair and other processes, relevant to anti-cancer effects. The anti-cancer activity of GHK may be linked to its known tissue remodeling effects [49].

### 2.10. Ubiquitin Proteasome System

The ubiquitin proteasome system (UPS) is a system that processes and clears damaged proteins. When this system is not functioning properly, damaged proteins may start accumulating. Aging is associated with decreased activity of the ubiquitin proteasome system. So far, there are no effective therapies to increase the UPS activity. Recent work has demonstrated that proteasome activation by either genetic means or use of compounds slows down aging [50].

We performed a search, using gene titles containing “ubiquitin” or “proteasome”. GHK strongly stimulates the gene expression of the UPS system, increasing activity of 41 genes and suppressing 1 gene. See Table 3.

According to Broad Institute data, UPS genes changed at least 50% UP or DOWN. GHK increased gene expression in 41 UPS genes while suppressing 1 UPS gene. This should have a positive effect on this system.

### 2.11. Anti-Pain, Anti-Anxiety, Anti-Aggression

GHK has potent anti-pain, anti-anxiety (anxiolytic) and anti-aggression actions.

Anti-pain effects were measured by determining how long it took for mice to lick their paws after being placed on a mildly-hot plate. Here, GHK reduced pain at a dose of 0.5 milligrams/kilogram. GHK has a physical structure similar to cimetidine which is often used to reduce pain in humans [51,52].

The anti-anxiety, anti-pain and anti-aggression actions were found in rats. When rats are afraid, they try to hide. But within 12 min of intraperitoneal injection of GHK at 0.5 micrograms/kilogram into rats in a testing cage built as a maze, the amount of time the rats spent exploring more open and lighted areas of the maze increased, and the time spent immobile (the freeze reaction) decreased, which indicated a reduction of fear and anxiety [53]. The same occurred in a test “open field”, where the rats spent less time hiding and more time exploring the area [54].

Likewise, the anti-aggression actions were found in rats. For the experiment, two rats are placed in a small cage and then given small electrical shocks, which produces anger in the rats and led to physical attacks on the other rat. The attacks were reduced 5-fold, when rats were placed into an agression-stimulating environment 12 min after the injection of 0.5 micrograms GHK per kilogram of body weight. If scaled up for human weight, this suggests that a similar effect might be induced in humans by 35 micrograms of GHK, which is a very low and safe dosage [55].

Studies of GHK passage through the skin by Howard Maibach’s laboratory suggest that it may be possible to easily pass an adequate amount of GHK-Cu through the skin to reduce anxiety, and possibly pain [56].

A manual search of genes affected by GHK found that seven anti-pain genes increased and two genes decreased. The results are presented in the Table 4. 

Psychological stress is an adaptive response that can be deleterious under certain conditions. Stress and anxiety delay epidermal barrier recovery, impair skin immune function, increase inflammation and oxidative stress. It causes activation of the hypothalamus-pituitary-adrenal (HPA) axis and the sympathetic nervous system. Skin is affected by molecules that are released during stress, such as neuropeptides, hormones, and cytokines [57]. This makes analgesic and anxiolytic effects of GHK an important part of its skin-related effects. Studies show that reducing anxiety and psychological stress can have a positive effect on chronic skin conditions such as psoriasis and atopic dermatitis [58]. Chronic stress can also impede wound healing, affecting recovery from plastic surgery and other invasive cosmetic procedures [59,60]. We believe that the GHK molecule evolved as the first response to an injury, and as such it is not surprising that it possesses analgesic and anxiolytic effects. Lowering the level of stress-related hormones and cytokines could help animals reduce inflammation and increase the chance of surviving the injury.

### 2.12. GHK Formulation and Delivery

GHK-Cu can penetrate the stratum corneum, which ensures its activity in cosmetic formulations [61,62]. However, to increase GHK’s penetration, it is advisable to use liposomes, including nanosized liposomes [63].

GHK is safe and very inexpensive. It can be easily incorporated into skin protective formulations, such as sunscreens and daytime creams and serums, as well as anti-wrinkle formulations. Because of its ability to improve wound healing, it is recommended after plastic surgery, chemical peels, dermabrasion, laser treatment, and so on. It will be very useful in a clinical setting and in assisted living senior facilities as a wound dressing, especially for diabetic and ischemic wounds.

Its safety record and its ability to reverse gene expression back to health warrant its use as a dietary supplement to support health and vitality of skin, hair and the entire body.

## 3. Conclusions

GHK is a small molecule, which possesses a surprisingly wide range of health-promoting qualities, while new studies are still revealing an even broader scope of GHK’s biological effects.

In the past, the wound healing, tissue remodeling, angiogenesis-promoting, cell-growth stimulating, anti-inflammatory and anti-oxidant actions of GHK were attributed to its unique relationship with copper. Copper is a transitional metal that is vital for all eukaryotic organisms from microbes to humans. Since it can be converted from oxidized Cu(II) to reduced Cu(I) form, it functions as an essential co-factor in a multitude of biochemical reactions involving electron transfer. A dozen enzymes (cuproenzymes) use changes in copper oxidation states to catalyze important biochemical reactions, including cellular respiration (cytochrome c oxidase), antioxidant defense (ceruloplasmin, superoxide dismutase (SOD), detoxification (metallothioneins), blood clotting (blood clotting factors V and VIII), and the connective tissue formation (lysyl peroxidase). Copper is required for iron metabolism, oxygenation, neurotransmission, embryonic development and many other essential biological processes [64].

Even though the copper hypothesis of GHK’s mode of action is still valid, we feel that it doesn’t explain the gene modulating effects of GHK-Cu. Therefore, in light of the new gene data, a new model of GHK-Cu action is needed, which will require collaboration of researchers from different fields.

As new gene profiling studies reveal, GHK with and without copper affects a large number of genes related to an organism’s response to stress and injury (tissue remodeling, anti-oxidant, anti-inflammatory, anti-pain, anti-anxiety, blood vessel growth, nerve outgrowth, anti-cancer action). GHK sequence is included in the collagen molecule, and SPARC protein and GHK is naturally released after an injury due to protein breakdown.

It is now known that some age-related changes in gene expression are not permanent and can be reversed. Studies show that regular physical exercise of older humans, as little as 30 min daily three times a week, can reset mitochondrial human DNA to a gene expression more like that of a younger person. Other procedures such as healthy diets, wine consumption, and flavonoid supplements are able to modify activity of certain genes, and various types of mediation and anti-stress methods are recommended to improve gene expression [65,66]. However, most biological compounds tested for their effects on gene expression using computer-based tools often lack supporting biological data. GHK has been extensively studied for over four decades and its safety and biological effects has been confirmed in cell, tissue and animal studies. In our opinion, the COPD study (Campbell et al.) is the most representative in this aspect, because not only were the gene signatures derived from areas with histologically confirmed pathology, but also GHK’s effects on affected lung tissue were tested in vitro and their correlation to gene effects was well-established [37].

GHK is a safe, inexpensive, extensively studied compound that has a wealth of positive and health-promoting effects in many tissues and systems. It has been widely used in anti-aging and cosmetic products in humans for decades without any adverse effects, and can be easily incorporated in creams, liposomes, dermal patches or delivered through microneedles. At present, it is not formulated into dietary supplements, so in our opinion, developing and testing GHK-based products for internal use to support health of elderly populations and as a complimentary therapy in cancer treatment is one possible direction for future research. Based on both biological and gene data, GHK also has the potential to be developed into an anti-anxiety and anti-pain supplemental treatment, and it may be an essential component in a future complex approach to COPD therapy.

## Figures and Tables

**Table 1:** Estimate of number of genes affected by glycyl-l-histidyl-l-lysine (GHK) [5].

Percent Change	Genes Stimulated	Genes Suppressed
50–99%	1569	583
100–199%	646	469
200–299%	227	196
300–599%	196	207
600–899%	39	47
900–1199%	8	7
1200% or more	2	4

**Table 2:** Genes Relevant to Neurons’ Function upregulated and downregulated by GHK.

Gene Title and Abbreviation (The GENE Database)	Percent Change
OPRM1, 1 opioid receptor, mu 1	+1294
TP73, 2 tumor protein p73	+938
KCND1, 3 potassium voltage-gated channel, Shal-related subfamily, member 1	+845
SLC8A2, 4 solute carrier family 8 (sodium/calcium exchanger), member 2	+737
CNTNAP2, 5 contactin associated protein-like 2	+581
STMN3, 6 stathmin-like 3	+500
LPHN3, 7 latrophilin 3	+494
ANGPT1, 8 angiopoietin 1	+487
SYN3, 9 synapsin III	+478
DPP6,10 dipeptidyl-peptidase 6	+448
PITX3, 221 paired-like homeodomain 3	−541
NOTCH3, 222 notch 3	−547
DLGAP1, 223 discs, large homolog-associated protein 1	−547
SLIT1, 224 slit homolog 1	−553
BSN, 225 bassoon (presynaptic cytomatrix protein)	−563
CELSR1, 226 cadherin, EGF LAG seven-pass G-type receptor 1	−647
CACNB4, 227 calcium channel, voltage-dependent, beta 4 subunit	−672
NDN, 228 necdin homolog (mouse)	−729
EDNRB, 229 endothelin receptor type B	−768
CHRM2, 230 cholinergic receptor, muscarinic 2	−1049

**Table 3:** GHK’s Effect on Gene Expression relevant to the Ubiquitin/Proteasome System [4].

	**Gene Title-Gene-Expression Increased**	**Percent Change**
1	ubiquitin specific peptidase 29, USP29	+1056
2	ubiquitin protein ligase E3 component n-recognin 2, UBR2	+455
3	gamma-aminobutyric acid (GABA) B receptor, GABBR1	+310
4	ubiquitin specific peptidase 34, USP34	+195
5	parkinson protein 2, E3 ubiquitin protein ligase (parkin), PARK2	+169
6	ubiquitin-conjugating enzyme E2I (UBC9 homolog, yeast), UBE2I	+150
7	ubiquitin protein ligase E3 component n-recognin 4, UBR4	+146
8	ubiquitin protein ligase E3B, UBE3B	+116
9	ubiquitin specific peptidase 2, USP2	+104
10	ubiquitin-like modifier activating enzyme 6, UBA6	+104
11	ubiquitination factor E4B (UFD2 homolog, yeast), UBE4B	+99
12	ubiquitin-conjugating enzyme E2M (UBC12 homolog, yeast), UBE2M	+92
13	Ubiquitin-like modifier activating enzyme 7, UBA7	+88
14	HECT, C2 and WW domain containing E3 ubiquitin protein ligase 1, HECW1	+81
15	proteasome (prosome, macropain) 26S subunit, ATPase, 3, PSMC3	+81
16	ubiquitin-conjugating enzyme E2D 1 (UBC4/5 homolog, yeast), UBE2D1	+79
17	proteasome (prosome, macropain) subunit, beta type, 2, PSMB2	+79
18	ubiquitin protein ligase E3 component n-recognin 5, UBR5	+77
19	ubiquitin specific peptidase 21, USP21	+76
20	OTU domain, ubiquitin aldehyde binding 2, OTUB2	+76
21	proteasome (prosome, macropain) inhibitor subunit 1 (PI31), PSMF1	+75
22	ubiquitin-conjugating enzyme E2H (UBC8 homolog, yeast), UBE2H	+73
23	ubiquitin-conjugating enzyme E2N (UBC13 homolog, yeast), UBE2N	+72
24	ubiquitin carboxyl-terminal hydrolase L5, UCHL5	+71
25	ubiquitin specific peptidase 6 (Tre-2 oncogene) pseudogene, LOC220594	+71
26	proteasome (prosome, macropain) 26S subunit, non-ATPase, 13, PSMD13	+70
27	ubiquitin associated protein 1, UBAP1	+70
28	ubiquitin-conjugating enzyme E2B (RAD6 homolog), UBE2B	+69
29	TMEM189-UBE2V1 readthrough, TMEM189-UBE2V1	+67
30	proteasome (prosome, macropain) 26S subunit, non-ATPase, 1, PSMD1	+64
31	proteasome (prosome, macropain) 26S subunit, non-ATPase, 3, PSMD3	+64
32	ariadne homolog, ubiquitin-conjugating enzyme E2 binding protein, 1 (Drosophila), ARIH1	+61
33	BRCA1 associated protein-1 (ubiquitin carboxy-terminal hydrolase), BAP1	+60
34	ubiquitin interaction motif containing 1, UIMC1	+60
35	ubiquitin associated protein 2-like, UBAP2L	+57
36	ubiquitin protein ligase E3 component n-recognin 7 (putative), UBR7	+56
37	ubiquitin-conjugating enzyme E2G 1 (UBC7 homolog, yeast), UBE2G1	+54
38	itchy E3 ubiquitin protein ligase homolog (mouse), ITCH	+54
39	ubiquitin-conjugating enzyme E2D 4 (putative), UBE2D4	+51
40	proteasome (prosome, macropain) 26S subunit, non-ATPase, 10, PSMD10	+50
41	WW domain containing E3 ubiquitin protein ligase 1, WWP1	+50
42	ubiquitin-like 3, UBL3	+50
	**Gene Title-Gene Expression Suppressed**	**Percent Change**
1	ubiquitin associated and SH3 domain containing A, UBASH3A	−89

**Table 4:** GHK and Genes Associated with Pain [30].

Gene Abbreviation, Name (GENE Database)	Percent Change	Comment (GENE Database)
ORPM1, opioid receptor mu 1	+1294	the principal target of endogenous opioid peptides such as beta-endorphin and enkephalins.
CCKAR, cholecystokinin A receptor	+190	regulates satiety and the release of beta-endorphin and dopamine.
CNR1, cannabinoid receptor 1	+172	involved in the cannabinoid-induced CNS effects
SIGMAR1, sigma non-opioid intracellular receptor 1	+155	a receptor protein that interacts with a variety of psychotomimetic drugs, including cocaine and amphetamines.
PNOC, prepronociceptin	+150	A receptor for proteins involved in regulation of pain sensitivity
OXT, oxytocin/neurophysin I prepropeptide	+136	A precursor protein for oxytocin
GRIA3, glutamate ionotropic receptor AMPA type subunit 3	−126	Glutamate receptors are the predominant excitatory neurotransmitter receptors in the mammalian brain
OPRK1, opioid receptor kappa 1	−119	a receptor for various synthetic opioids
